# Headache-attributed burden among children and adolescents in Georgia: estimates from a national cross-sectional schools-based study

**DOI:** 10.1186/s10194-026-02451-7

**Published:** 2026-07-31

**Authors:** Nana Nino Tatishvili, Zaza Katsarava, Teona Shatirishvili, Tamar Kipiani, Tinatin Giuashvili, Mariam Lomsianidze, Aleksandre Kotetishvili, Anano Kvernadze, Irakli Tugushi, Salome Maghlakelidze, Vera Nemsadze, Ana Bedoshvili, Tamar Bitskinashvili, Giorgi Sakvarelidze, Derya Uludüz, Tayyar Şaşmaz, Deniz Erdal, Andreas Kattem Husøy, Timothy J Steiner

**Affiliations:** 1 David Tvildiani Medical University (DTMU), Tbilisi, Georgia; 2 Centre of Neurology, Geriatric Medicine and Early Rehabilitation, Evangelical Hospital, Unna, Germany; 3 Medical Faculty, University of Duisberg-Essen, Essen, Germany; 4 Neuroscience Department, M Iashvili Central Children’s Hospital, Tbilisi, Georgia; 5 Department of Neurology, Faculty of Medicine, Istanbul Atlas University, Istanbul, Turkey; 6 Department of Public Health, Mersin University School of Medicine, Mersin, Turkey; 7 Department of Neuromedicine and Movement Science, Norwegian Centre for Headache Research (NorHead), Norwegian University of Science and Technology, Trondheim, Norway; 8 Department of Neurology and Clinical Neurophysiology, St Olavs University Hospital, Trondheim, Norway; 9 Department of Neurology, University of Copenhagen, Copenhagen, Denmark; 10 Division of Brain Sciences, Imperial College London, London, UK; 11 Department of Neuromedicine and Movement Science, Norwegian University of Science and Technology (NTNU), Edvard Griegs gate 8, Trondheim, Norway

**Keywords:** Headache disorders, Disease definition, Burden of disease, Children, Adolescents, Schools-based survey, Georgia, European region, Global Campaign against Headache

## Abstract

**Background:**

We recently published an account of headache prevalence among children (6–11 years) and adolescents (12–17) in Georgia, reporting an age- and gender-adjusted 1-year estimate of 66.3% for all headache. With undifferentiated headache (UdH) defined as mild or moderate headache lasting < 1 h, age- and gender-adjusted prevalence of migraine was 22.2%, of tension-type headache (TTH) 9.5%, of UdH 30.2%, of probable medication-overuse headache (pMOH) 0.5%, and of other headache on ≥ 15 days/month (other H15+) 3.6%. Here, using the same definition of UdH, we present data on attributed burden, collected contemporaneously from the same participants. The study was within the worldwide programme of cross-sectional schools-based studies undertaken by the Global Campaign against Headache.

**Methods:**

We used the Global Campaign’s standardized protocol, selecting schools representative of the country. The child and adolescent versions of the structured Headache-Attributed Restriction, Disability, Social Handicap and Impaired Participation (HARDSHIP) questionnaire, translated into Georgian language, were completed by pupils under supervision in class. Headache diagnoses followed International Classification of Headache Disorders, edition 3 (ICHD-3), except for UdH, which was defined as mild or moderate headache lasting < 1 h. Burden enquiry was in multiple domains, including symptom burden, impaired participation and quality of life (QoL), with timeframes of 4 weeks and 1 day (the latter based on headache yesterday [HY]).

**Results:**

Symptom burden was evidenced by mean headache frequency of 4.3 days/4 weeks, duration 1.6 h, proportion of time in ictal state 1.5% and intensity 1.7 (mild to moderate). Of episodic headaches, migraine was the most frequent, longest lasting and most intense. Pupils with pMOH reported headache on 78.9% of all days. Except for pMOH, fewer than half of headache days were medicated. Headache was blamed for 0.7 days/4 weeks of missed school (3.5% of 20 days), although only 1.5% of the 1,910 pupils with headache in the last year were absent yesterday because of HY (this analysis could not capture lost days immediately preceding weekends or holidays, so underestimated by at least 20%). Almost one in five parents (18.3%) of children lost time from their own work because of their child’s headache, but only 4.5% of parents of adolescents. Emotional impact and QoL scores both showed gradation across headache types (pMOH and other H15 + worse than migraine worse than TTH and UdH).

**Conclusions:**

Headache among children and adolescents in Georgia is associated with individual- and population-level burdens that measurably and adversely affect health, wellbeing, education and participation in leisure activities. These findings are available to inform national education and health policies.

## Introduction

We recently reported estimates of headache prevalence among children (6–11 years) and adolescents (12–17) in Georgia [1], an upper-middle-income country in Eastern Europe with an autochthonous population. We found two thirds (68.5%) of participants (*N* = 2,790) reported headache in the preceding year (age- and gender-adjusted 1-year prevalence: 66.3%). Headache yesterday (HY) (1-day prevalence) was reported by 20.4%.

The study included separate prevalence estimates for migraine, tension-type headache (TTH) and undifferentiated headache (UdH), as well for probable medication-overuse headache (pMOH) and other headache on ≥ 15 days/month (other H15+). However, in studies using the same methodology in Nepal [2], Benin [3] and Zambia [4], the conventional definition of UdH (mild headache lasting < 1 h [5]) had been questioned, and a modified definition proposed (*mild or moderate* headache lasting < 1 h). Our study in Georgia therefore made estimates according to each: by the conventional definition, 1-year prevalence of UdH was 18.2%; by the modified definition it was 30.2% [1]. Since UdH took diagnostic precedence over migraine and TTH, estimates for these were dependent on the definition of UdH: for migraine (definite plus probable) 29.1% with UdH defined conventionally, reduced to 22.2% with the modified definition, and for TTH (definite plus probable) 13.1%, reduced to 9.5%. Estimates for pMOH (0.5%) and other H15+ (3.6%) were unaffected by the definition of UdH [1].

We argued that there was convincing evidence from these studies for adopting the modified criteria for UdH [1–4]. Here we make estimates of burden attributed to headache overall and to each headache type, deriving these in accordance with diagnoses and prevalence estimates based on the modified definition of UdH [1].

Burden estimates are much more informative to health and education policies in Georgia than those of prevalence. However, all contribute to the Global Burden of Disease (GBD) study [6], to our understanding of headache among these age groups, and to our knowledge of the global burden of headache disorders.

## Methods

This was a cross-sectional, schools-based survey. The detailed methodology has been published previously [1, 7], so we present only a summary here. Data collection took place between December 2022 and November 2023, at the same time and from the same participants as the prevalence data.

### Ethics and approvals

The study was performed in accordance with the Declaration of Helsinki.

The protocol was approved in 2019 by David Tvildiani Medical University Ethics Committee (N6, 4.12.2019). Data collection was delayed by the COVID-19 pandemic, but completed in 2022/2023 under this approval. Because of the long delay, approval was re-affirmed in 2024 (N4, 23.02.2024).

Permission to perform this study was granted by the Georgian Ministry of Education. Additionally, permissions were obtained from academic and administrative authorities of each school, along with the consents of principals and class teachers.

All pupils participating in the study were informed of its nature and purpose, and gave oral consent before enrolment. Continuing consent was evidenced by completion of the questionnaires. Since the study was wholly innocuous, the ethics committee agreed that prior parental consent was not additionally needed.

### Study procedures

We used the standardized protocol for the worldwide series of schools-based studies developed by the Global Campaign against Headache [7]. We selected nine schools in regions distributed from west to east of the country: five urban (four in Tbilisi, the capital city, towards the east; one in Batumi, on the Black Sea [western] coast) and one in each of four rural regions (Chakvi in the west, Tserovani towards the north, Borjomi in the south, and Telavi in the east). We could not include the regions occupied by Russia (Abkhazia and Tskhinvali [South Ossetia]).

In mediated sessions within their classes, pupils aged 6–17 years who were present on the survey days completed the child and adolescent versions of the structured Headache-Attributed Restriction, Disability, Social Handicap and Impaired Participation (HARDSHIP) questionnaire [7], translated into Georgian language following the translation protocol for lay documents developed by *Lifting The Burden* (LTB) [8]. HARDSHIP included demographic enquiry, headache diagnostic questions based on ICHD-3 [9] (other than for UdH), and burden enquiry (symptom burden, medication use, impaired participation [lost time from school and other activities], lost parental worktime, emotional impact and quality of life [QoL]). The timeframes of enquiry were 4 weeks and 1 day, the latter based on HY.

### Analysis

Headache frequency was reported as days/4 weeks, headache intensity as “mild”, “moderate” or “severe” (which we converted to a numerical scale of 1–3, treated as continuous). Duration of headache episodes was reported in hours as < 1, 1–2, 2–4 or > 4, which we analysed by taking the mid-points (0.5, 1.5, 3 and 8 respectively, assuming a range of 4–12 h for the last). Acute medication intake was reported as days/4 weeks, regardless of quantity. We calculated proportion (%) of time in ictal state (pTIS) as the product of frequency and duration, and estimated lost health at individual level (%) as pTIS*DW, where DW, on a scale 0–1, was the disability weight attributed in the Global Burden of Disease (GBD) study to the ictal state of the headache type (migraine, TTH or pMOH) (0 indicating no health loss and 1 a health loss equivalent to being dead) [10].

We estimated lost school time in the preceding 4 weeks, counting not going to school as a whole day lost, and leaving school early as a half-day lost. We calculated proportions of days lost using a denominator of 20. We calculated proportions who had missed school yesterday because of HY, and, for comparison, proportions predicted to have missed school yesterday (the product of number affected by headache and mean reported days [divided by 20] lost per pupil over the preceding 4 weeks). We counted days of limited activity in the preceding 4 weeks (defined as “could not do things you wanted to because of your headaches”), presuming a denominator of 28. We counted parents of participating pupils who had, according to the pupils, lost time from their own work (regardless of quantity) during the same period. Responses to questions addressing concentration, emotional impact and QoL were all on a 4-point Likert scale (“never”, “sometimes”, “often”, “always”) [6], also referring to the preceding 4 weeks. We scored these 0–3, and summed them to generate an emotional impact score (including concentration; potential range 0–18, high being adverse) and a QoL score (0–36, low being adverse).

In descriptive statistics we used means, standard deviations (SDs) and standard errors (SEs) for continuous variables and proportions (%) with 95% confidence intervals (CIs) for categorical data. We used Kruskal-Wallis with post hoc Dunn’s test to evaluate group-differences for continuous variables, and binary logistic regression to evaluate group-differences for binary outcomes.

Population-based estimates of burden were derived by factoring in prevalence.

In all analyses, we set significance at *p* < 0.05.

## Results

### Sample description

Of 2,798 potential participants (pupils present on the day), six did not participate, two were excluded because of incomplete diagnostic data, and 2,790 (males 48.9%; females 51.1%; children 50.6%; adolescents 49.4%) entered the analysis (participating proportion 99.7%) [1].

Of these, 1,910 (843 males, 1,067 females; 755 children, 1,155 adolescents) reported headache of any type in the preceding year [1], and 568 reported HY. With UdH defined as mild or moderate headache lasting < 1 h, age- and gender-adjusted prevalence of migraine was 22.2%, of TTH 9.5%, of UdH 30.2%, of pMOH 0.5% and of other H15 + 3.6%. Only 1.7% remained unclassified. These proportions, published previously [1], are repeated here since they are used in the following analyses.

### Burden

Table 1 shows symptom burden. Headache frequency was very skewed by H15+, but the mean for all headache was 4.3 days/4 weeks, with a duration of 1.6 h. Mean pTIS was 1.5%. Mean intensity was 1.7 (mild to moderate). Of the episodic headaches, migraine was the most frequent, longest lasting (therefore associated with the highest pTIS) and most intense. Participants with pMOH reported headache on more than three quarters (78.9%) of all days, those with other H15 + on two thirds (65.0%). With durations of about 3.5 h, mean pTIS for each of these disorders was much higher: 11.8% and 10.0% respectively. Both pMOH and other H15 + were reportedly as intense as migraine (moderate) (Table 1).

**Table 1:** Symptom burden by headache type and overall

Headache type	Pupils affected (n)	Headache frequency days/4 weeks (mean±SD)	Headache duration hours (mean±SD)	Estimated pTIS % (mean±SE)	Headache intensity scale 1–3^1^ (mean±SD)
Episodic headaches					
Migraine	640	4.4±3.2	2.7±2.3	1.9±0.1	2.1±0.6
TTH	272	3.4±2.9	2.0±1.7	1.0±0.1	1.6±0.6
UdH	874	2.5±2.5	0.5± < 0.1	0.2± < 0.1	1.4±0.5
**Headache on ≥ 15 days/month**					
pMOH	13	22.1±4.4	3.6±3.2	11.8±3.0	2.3±0.6
Other H15+	104	18.2±4.2	3.5±2.9	10.0±0.9	2.1±0.6
**All headache** ^**2**^	1,910	4.3±4.8	1.6±2.0	1.5±0.1	1.7±0.6

SD: standard deviation; SE: standard error; pTIS: proportion (%) of time in ictal state; TTH: tension-type headache; UdH: undifferentiated headache; pMOH: probable medication-overuse headache; H15+: headache on ≥ 15 days/month; ^1^equating 1–3 to the reported categories “mild”, “moderate” and “severe”, and treating as continuous data; ^2^includes unclassified headache

Table 2 shows frequency of symptomatic medication use, along with frequency of headache (from Table 1) for comparison. The principal finding here was that, with the exception of pMOH, fewer than half of headache days were medicated. For other H15+, fewer than one quarter were.

Table 3 shows the health loss associated with each headache type at individual and population levels. This measure was estimated as the product of pTIS and DW for the ictal state, and therefore could be derived only for migraine, TTH and pMOH, to which DWs had been allocated in GBD [10]. At individual level, by far the greatest health loss was attributable to pMOH (2.6%), three times that attributable to migraine (0.8%). At population level, however, with prevalence a factor, only migraine had appreciable impact (0.19%).

Table 4 shows impaired participation, assessed as lost time from school and from other activities (social, leisure and play). Among those affected, all headache was responsible for 0.7 days of missed school each 4 weeks, a loss of 3.5% assuming a 5-day week, with little difference between children and adolescents. Days of limited activities were twice as many (1.4), but with a presumed denominator of 28 days (5.0%). pMOH had by far the most serious impact: 5.0 days/4 weeks lost from school equated to one quarter (25.0%) of all school time, while 8.0 days of limited activities equated to 28.6% of all days.

**Table 2:** Symptomatic medication use by headache type and overall

Headache type	Pupils responding n	Medication use days in preceding 4 weeks (mean ± SD)	Headache frequency days/4 weeks (mean ± SD) (from Table 1)
**Episodic headaches**			
Migraine	640	2.0±2.1	4.4±3.2
Tension-type headache	272	1.3±1.6	3.4±2.9
Undifferentiated headache	874	1.0±1.5	2.5±2.5
**Headache on ≥ 15 days/month**			
Probable medication-overuse headache	13	20.6±3.7	22.1±4.4
Other headache on ≥ 15 days/month	104	4.2±3.6	18.2±4.2
**All headache** ^**1**^	1,910	1.7±2.6	4.3±4.8

^1^Includes unclassified headache

**Table 3:** Headache-attributed lost health by headache type

Headache type	Disability weight (from [10])	Age- and gender adjusted prevalence (from [1]) %	Mean pTIS (from Table 1) %	Lost health %	
				Individual^1^ mean	Population^2^
Migraine	0.441	22.1	1.9	0.8	0.19
Tension-type headache	0.037	9.7	1.0	0.04	0.004
Probable medication-overuse headache	0.223	0.5	11.8	2.6	0.01

pTIS: proportion of time in ictal state; ^1^calculated as the product of mean pTIS and disability weight; ^2^calculated as the product of mean individual lost health and prevalence (with apparent discrepancies due to rounding)

Table 5 shows lost school time assessed from absences yesterday attributed to HY, a more objective and arguably more reliable measure. The differences between actual absences and predicted (from prevalence and mean reported lost days/4 weeks) showed an apparent tendency to overestimation. For example, the actual probability of a missed school day yesterday for a pupil with migraine was 2.3% (15/640), about half the predicted probability (29/640 = 4.5%). The differences were greater for TTH and UdH (numbers with pMOH were very low). For all headache, the actual probability was 1.5% (29/1,910), less than half the predicted (67/1,910 = 3.5%). There is commentary on this finding in the Discussion.

**Table 4:** Lost time from school and other activities because of headache in the preceding 4 weeks, by headache type and overall, and by age group

Headache type	Headache frequency (from Table 1) days/4 weeks (mean)	Days of lost school	Days of limited activity
		days/4 weeks/affected pupil (mean ± SD)	
**Episodic headaches**			
Migraine children adolescents	4.4±3.2 4.1±3.1 4.6±3.3	0.9±1.4 1.1±1.5 0.8±1.3	1.8±2.3 1.9±2.4 1.8±2.2
Tension-type headache children adolescents	3.4±2.9 2.9±2.5 3.6±3.0	0.6±1.2 0.7±1.1 0.6±1.2	1.0±1.5 1.2±1.6 0.9±1.4
Undifferentiated headache children adolescents	2.5±2.5 2.3±2.5 2.7±2.6	0.5±1.0 0.5±1.0 0.4±1.0	0.7±1.5 0.8±1.5 0.7±1.5
**Headache on ≥ 15 days/month**			
Probable medication-overuse headache children adolescents	22.1±4.4 21.3±5.5 22.3±4.4	5.0±4.3 4.8±5.6 5.1±4.1	8.0±6.1 6.0±1.7 8.6±6.9
Other headache on ≥ 15 days/month children adolescents	18.2±4.2 20.2±4.8 17.7±3.9	1.8±2.3 2.1±2.4 1.7±2.3	5.1±4.9 4.4±5.4 5.3±4.7
**All headache** ^**1**^ children adolescents	4.3±4.8 3.5±4.2 4.8±5.1	0.7±1.4 0.8±1.3 0.7±1.4	1.4±2.4 1.3±2.2 1.5±2.6

^1^Includes unclassified headache

Table 6 shows numbers of parents who lost time in the preceding 4 weeks (regardless of amount) from their own work because of a son’s or daughter’s headache. Rather fewer than one in ten parents (9.4%) lost at least some time, but four-fold more parents of children (18.3%) than of adolescents (4.5%). A similar differential was seen for all episodic headache types. Numbers were very small for pMOH.

**Table 5:** Lost school days yesterday because of headache yesterday (HY), by headache type and overall, with predictions based on recalled lost days/4-weeks

Diagnosed headache type	Pupils affected n	Proportion reporting HY n (% of those with headache type)	Missed school day yesterday n (% of those reporting HY)	
			Actual	Predicted
**Episodic headaches**				
Migraine	640	242 (37.8)	15 (6.2)	29 (12.0)
Tension-type headache	272	71 (26.1)	3 (4.2)	8 (11.3)
Undifferentiated headache	874	144 (16.5)	4 (2.8)	22 (15.3)
**Headache on ≥ 15 days/month**				
Probable medication-overuse headache	13	11 (84.6)	1 (9.1)	3 (27.3)
Other headache on ≥ 15 days/month	104	99 (95.2)	6 (6.1)	9 (9.1)
**All headache** ^**1**^	1,910	568 (29.7)	29 (5.1)	67 (11.8)

^1^Includes unclassified headache

The measures of impaired participation (lost time from school and from other activities, and lost parental worktime) are compared statistically in Table 7, with the purpose of determining how well these measures distinguished between diagnoses – particularly migraine from TTH and UdH. The first two measures did so clearly. The third distinguished migraine from UdH but not from TTH, but also, with low numbers for pMOH and other H15+, failed to distinguish between migraine and H15+.

Table 8 (which includes similar between-type comparisons) shows emotional impact and QoL scores, the latter also for those with no headache. Emotional impact was greatest (highest scores) for pMOH (8.8) and other H15+ (8.5), and greater for migraine (6.9) than for TTH (5.3) or UdH (4.9). Baseline QoL was low (28.5 out of the possible total of 36 for those with no headache), but all headache types nevertheless had significant adverse impact upon QoL. Again, impact was greatest (lowest scores) for pMOH and other H15+ (both scoring around half of the possible total), and greater for migraine (21.4) than for TTH (23.9) or UdH (24.7).

**Table 6:** Parents’ lost work in the preceding 4 weeks, by pupils’ headache type and overall, and by age group

Headache type	Pupils affected n	Headache frequency days/4 weeks (from Table 1)	Parent missed work n (% of pupils with headache type)
**Episodic headaches**			
Migraine children adolescents	640 223 417	4.4±3.2 4.1±3.1 4.6±3.3	77 (12.0) 52 (23.3) 25 (6.0)
Tension-type headache children adolescents	272 88 184	3.4±2.9 2.9±2.5 3.6±3.0	22 (8.1) 17 (19.3) 5 (2.7)
Undifferentiated headache children adolescents	874 415 459	2.5±2.5 2.3±2.5 2.7±2.6	68 (7.8) 53 (12.8) 15 (3.3)
**Headache on ≥ 15 days/month**			
Probable medication-overuse headache children adolescents	13 3 10	22.1±4.4 21.3±5.5 22.3±4.4	4 (30.8) 3 (100.0) 1 (10.0)
Other headache on ≥ 15 days/month children adolescents	104 21 83	18.2±4.2 20.2±4.8 17.7±3.9	9 (8.7) 3 (14.3) 6 (7.2)
**All headache** ^**1**^ children adolescents	1,910 755 1,155	4.3±4.8 3.5±4.2 4.8±5.1	180 (9.4) 128 (18.3) 52 (4.5)

^1^Includes unclassified headache

**Table 7:** Comparisons of headache types for measures of impaired participation (Kruskal-Wallis with post-hoc Dunn’s test)

Lost school days/4 weeks						
	*n* ^1^	mean±SD	TTH	UdH	pMOH	Other H15+
Migraine	640	0.9±1.4	**0.001**	**< 0.001**	**< 0.001**	**0.02**
TTH	272	0.6±1.2		0.09	**< 0.001**	**< 0.001**
UdH	874	0.5±1.0			**< 0.001**	**< 0.001**
pMOH	13	5.0±4.3				**0.02**
Other H15+	104	1.8±2.3				
**Lost days/4 weeks from other activities**						
Migraine	640	1.8±2.3	**< 0.001**	**< 0.001**	**< 0.001**	**< 0.001**
TTH	272	1.0±1.5		**0.02**	**< 0.001**	**< 0.001**
UdH	874	0.7±1.5			**< 0.001**	**< 0.001**
pMOH	13	8.0±6.1				0.24
Other H15+	104	5.1±4.9				
**Lost parental worktime** n^1^ (%) of parents of pupils with headache type reported to have lost time in preceding 4 weeks						
Migraine	77	12.0%	0.41	0.05	0.31	0.86
TTH	22	8.1%		1.00	0.08	1.00
UdH	68	7.8%			0.05	0.99
pMOH	4	30.8%				0.17
Other H15+	9	8.7%				

TTH: tension-type headache; UdH: undifferentiated headache; pMOH: probable medication-overuse headache; H15+: headache on ≥ 15 days/month; ^1^values reflect that not all pupils responded to every enquiry; significant p-values are emboldened

**Table 8:** Emotional impact and quality of life scores by headache type

Headache type	Pupils responding n^1^	Score mean ± SD	Between-type significance (Kruskal-Wallis with post-hoc Dunn’s test)			
			TTH	UdH	pMOH	Other H15+
**Emotional impact** (0–18; higher scores adverse)						
Migraine	640	6.9±2.9	**< 0.001**	**< 0.001**	0.32	**< 0.001**
TTH	272	5.3±3.0		0.40	**0.001**	**< 0.001**
UdH	874	4.9±2.8			**< 0.001**	**< 0.001**
pMOH	13	8.8±3.2				1.00
Other H15+	104	8.5±4.0				
All headache^2^	1,910	5.9±3.1				
**Quality of life** (0–36; lower scores adverse)						
No headache	880	28.5±4.7	**< 0.001** against all headache types			
Migraine	640	21.4±5.9	**< 0.001**	**< 0.001**	0.16	**< 0.001**
TTH	272	23.9±5.4		0.28	**0.002**	**< 0.001**
UdH	874	24.7±5.6			**< 0.001**	**< 0.001**
pMOH	13	17.6±5.3				1.00
Other H15+	104	18.6±5.7				
All headache^2^	1,910	23.1±6.0				

TTH: tension-type headache; UdH: undifferentiated headache; pMOH: probable medication-overuse headache; headache on ≥ 15 days/month; ^1^values reflect that not all pupils responded to every enquiry; ^2^includes unclassified headache; significant p-values are emboldened

## Discussion

This schools-based study among children and adolescents in Georgia adds data on attributed burden to our previous study reporting headache prevalences [1]. These data, which we now summarise, are far more informative to health and educational policies in Georgia, while adding to knowledge and understanding of headache disorders among these age groups. Symptom burden (with data greatly skewed by H15+) was evidenced by mean headache frequency of 4.3 days/4 weeks, mean duration of 1.6 h, mean pTIS of 1.5% and mean intensity of 1.7 (mild to moderate). Of the episodic headaches, migraine was the most frequent, longest lasting and most intense. Pupils with pMOH reported headache on 78.9% of all days. With the exception of pMOH, fewer than half of headache days were medicated. Headache was blamed for 0.7 days of missed school each 4 weeks (3.5% of 20 days), although only 29 pupils (1.5% of the 1,910 with any headache in the last year) were absent because of HY on the day prior to the survey. However, the analysis based on HY would have underestimated lost school days by at least 20% because the survey could not capture lost days immediately preceding weekends or school holidays. Almost one in five parents (18.3%) of children lost at least some time from their own work because of their child’s headache, but only 4.5% of parents of adolescents. Emotional impact and QoL scores both showed gradation across headache types (pMOH and other H15+ worse than migraine; migraine worse than TTH and UdH).

In the earlier prevalence study [1], diagnostic uncertainties arose, as they had in studies elsewhere – in Nepal [2], Benin [3] and Zambia [4}. These have been discussed elsewhere at length [1–4]: the principal uncertainty arises from the distinction – often depending solely on reported headache intensity – between probable migraine and UdH (the latter believed to be expressions by the immature brain of what will later develop into migraine or TTH [5]). We sought to resolve this by redefining UdH to include *mild and moderate* headache, retaining the crucial characteristic of very short duration (< 1 h) [1–4]. It is reassuring, therefore, that, with this revised definition, most burden measures, particularly lost time from school and from other activities, and the emotional impact and QoL scores, clearly distinguished migraine from UdH (and from TTH).

How heavy are these burdens? Despite its short duration and mild-to-moderate intensity, headache on an average of >1 day/week during childhood and adolescence is likely to be perceived as a significant loss of health and wellbeing. This is clearly evidenced by the QoL scores: even with a low baseline of 28.5/36 (those with no headache), these scores showed considerable sensitivity to headache, being reduced by migraine to 21.4 and by pMOH to 17.6. Lost school time is always an important burden because it has potential adverse consequences over the lifetime. Overall, the losses among all pupils with headache might be less than 2%, but, as in Benin [3], those with pMOH reportedly lost a quarter of their schooling, and a slightly greater proportion of their time from other activities (which is likely to affect social development). Fortunately, pMOH was uncommon (0.5% [1]), while other H15+, although more prevalent at 3.6% [1], was mostly unmedicated and not, therefore, an obvious risk factor for developing pMOH.

Only two studies have made comparable analyses of headache-attributed burden (i.e., with the modified criteria for UdH), both being Global Campaign studies using the same methodology. Both were from countries in sub-Saharan Africa (Benin [3] and Zambia [4]), very different from Georgia in terms of geography, ethnicity, culture, climate, and organisation and availability of health care. Nevertheless, there are some similarities in the findings. Burden estimates built upon revised prevalence estimates (with the modified criteria for UdH) were broadly similar between the studies in Benin and Zambia, with rather lower symptom burden in the former [3]. In Georgia we found the same average headache frequency as in Benin (4.3 *versus* 4.2 days/4 weeks [3]), somewhat milder headache (1.7 *versus* 2.1 [3], but shorter reported mean duration (1.6 *versus* 2.8 h [3]) and therefore lower mean pTIS (1.5% *versus* 2.0% [3]). However, in Zambia, pTIS was 1.2%, with lower frequency of 2.9 days/4 weeks but mean duration of 2.3 h [4]. Medication days were fewer than headache days in all three countries. Pupils with headache in Georgia were much less likely to miss school (5.1% of those with HY) than those in Benin (15.8% [3]) or Zambia (13.8% [4]), but Benin in particular is notable for very high levels of school absenteeism from all causes (cultural as well as medical) [11]. Fewer parents in Georgia (9.4%) lost time from their work than in Benin (30.3% [3]) or Zambia (15.9% [4]). Many factors might have operated here, but in all cases there were higher losses among parents of children than parents of adolescents, a reflection, almost certainly, of increasing independence among adolescents, not of decreasing illness.

In all three studies, measures of impaired participation, and emotional impact and QoL scores, clearly distinguished between migraine and UdH (defined, essentially, by duration < 1 h). In all three studies we have concluded that pupils diagnosed as having migraine were reporting a headache measurably and consistently more burdensome than those diagnosed as having UdH, supporting the belief that the latter requires separate classification.

The findings from this study are available to inform health and education policies in Georgia. Emerging from 70 years of Soviet occupation in 1991 [12], Georgia does not have a history of good headache care, or of political will to introduce it [13]. But change is possible. In a population-based study in 2009, we found the prevalence of migraine in Georgia (15.6% [14]) to be in line with the global average estimated in GBD 2010 (14.7% [15]), while the prevalence of H15+ (7.6% [14]) – which might include chronic migraine, chronic TTH and MOH – matched the average for (mainly Western) Europe (7.1%) estimated in the Eurolight project [16]. In other words, Georgia had no less headache than elsewhere, but no health-care programme for it [13]. In the following years, following a willingness-to-pay enquiry [17], we trained doctors and helped establish several headache clinics [13], which continue both to serve people with headache in Georgia (albeit mainly in Tbilisi) according to international standards [18] and to participate in European headache research [19]. Now, Georgia is ranked as an upper-middle-income country [20]. Its health policy should, therefore, consider how this evidence – not only of ill health among its paediatric population but also of the long-term damaging consequences of lost school time associated with it – should influence its priority-setting. At the very least, a health-care needs assessment should be undertaken, focusing on those with migraine or H15+ (especially the latter). With regard to lost schooling and educational policy, an average of 0.7 days/4 weeks of lost school time may or may not be considered important, but the 9–25% of school time lost by those with H15 + certainly is. Only 4% of participants had H15+, but their needs probably include remedial teaching as well as health care.

Limitations of this study, identified previously [1], include the general limitations of all surveys among young people arising from necessary dependence on their understanding, recall and ability to report reliably. We mitigated the first through mediated enquiry, and the second by additional enquiry into HY. We did not attempt to diagnose other H15+, which, within an overall prevalence of 3.6% [1], might have included some cases of chronic migraine or chronic TTH. To the extent that this happened, burdens attributed to migraine and TTH were underestimated. The strengths of the study lay in the tested and validated methodology [7], adequate sample size and very high participating proportion (99.7% [1]) – the last being a major benefit of schools-based sampling. Very few headaches were unclassified (1.7% [1]).

## Conclusions

Headache among children and adolescents in Georgia is associated with individual- and population-level burdens that measurably and adversely affect health, wellbeing, education and participation in social and leisure activities. These findings are of relevance to education and health policies in Georgia.

## Data Availability

The original data are held at David Tvildiani Medical University, and the analytical set at University of Mersin and at Norwegian University of Science and Technology. When analyses are completed, anonymised data will be available on request for academic purposes, in line with the policy of the Global Campaign against Headache.

## Abbreviation

CI: Confidence interval
DTMU: David Tvildiani Medical University
DW: Disability weight
GBD: Global Burden of Disease (study)
H15+: Headache on ≥15 days/month
HARDSHIP: Headache-attributed restriction, disability, social handicap and impaired participation (questionnaire)
HY: Headache yesterday
ICHD: International Classification of Headache Disorders
LTB: Lifting The Burden
MOH: Medication-overuse headache
NorHead: Norwegian Centre for Headache Research
NTNU: Norwegian University of Science and Technology
pMOH: Probable MOH
pTIS: Proportion of time in ictal state
QoL: Quality of life
SD: Standard deviation
SE: Standard error
TTH: Tension-type headache
UdH: Undifferentiated headache
